# Symptoms in unilateral vestibular hypofunction are associated with number of catch-up saccades and retinal error: results from the population-based KORA FF4 study

**DOI:** 10.3389/fneur.2023.1292312

**Published:** 2023-11-28

**Authors:** Maria Aubele, Eva Grill, Thomas Eggert, Erich Schneider, Ralf Strobl, Klaus Jahn, Martin Müller, Rolf Holle, Birgit Linkohr, Margit Heier, Karl-Heinz Ladwig, Nadine Lehnen

**Affiliations:** ^1^Department of Neurology, University Hospital, Ludwig-Maximilians-Universität München, Munich, Germany; ^2^German Center for Vertigo and Balance Disorders, University Hospital, Ludwig-Maximilians-Universität München, Munich, Germany; ^3^Department of Psychosomatic Medicine and Psychotherapy, University Hospital Rechts der Isar, Technical University of Munich, Munich, Germany; ^4^Institute for Medical Information Processing, Biometry and Epidemiology, Faculty of Medicine, Ludwig-Maximilians-Universität München, Munich, Germany; ^5^Institute of Medical Technology, Brandenburg University of Technology Cottbus-Senftenberg, Cottbus, Germany; ^6^Department of Neurology, Schoen Clinic Bad Aibling, Bad Aibling, Germany; ^7^Nursing Science and Interprofessional Care, Department for Primary Care and Health Services Research, Medical Faculty, Heidelberg University, Heidelberg, Germany; ^8^Institute of Epidemiology, Helmholtz Zentrum München, German Research Center for Environmental Health, Neuherberg, Germany; ^9^KORA Study Center, University Hospital Augsburg, Augsburg, Germany

**Keywords:** video head impulse test, vHIT, catch-up saccades, unilateral vestibular hypofunction, retinal error, vestibulo-ocular reflex, VOR, population-based survey

## Abstract

**Objective:**

The presence and intensity of symptoms vary in patients with unilateral vestibular hypofunction. We aimed to determine which saccadic and vestibulo-ocular reflex parameters best predict the presence of symptoms in unilateral vestibular hypofunction in order to better understand vestibular compensation and its implications for rehabilitation therapy.

**Methods:**

Video head impulse test data were analyzed from a subpopulation of 23 symptomatic and 10 currently symptom-free participants with unilateral vestibular hypofunction, embedded in the KORA (Cooperative Health Research in the Region of Augsburg) FF4 study, the second follow-up of the KORA S4 population-based health survey (2,279 participants).

**Results:**

A higher number of catch-up saccades, a higher percentage of covert saccades, and a larger retinal error at 200 ms after the onset of the head impulse were associated with relevant symptoms in participants with unilateral vestibular hypofunction (*p* = 0.028, *p* = 0.046, and *p* = 0.038, respectively). After stepwise selection, the number of catch-up saccades and retinal error at 200 ms remained in the final logistic regression model, which was significantly better than a null model (*p* = 0.014). Age, gender, saccade amplitude, saccade latency, and VOR gain were not predictive of the presence of symptoms.

**Conclusion:**

The accuracy of saccadic compensation seems to be crucial for the presence of symptoms in unilateral vestibular hypofunction, highlighting the role of specific gaze stabilization exercises in rehabilitation. Early saccades, mainly triggered by the vestibular system, do not seem to compensate accurately enough, resulting in a relevant retinal error and the need for more as well as more accurate catch-up saccades, probably triggered by the visual system.

## 1 Introduction

Unilateral vestibular hypofunction has many possible causes and can be abrupt or gradual in onset. In severe cases, symptoms range from vertigo and oscillopsia to postural unsteadiness, ataxia, and falls (1). Although most patients recover over time, some continue to experience symptoms of dizziness or imbalance and report lower health-related quality of life (2). Especially for vestibular rehabilitation, a mainstay of therapy for unilateral vestibular hypofunction (3), it is important to know why some patients experience symptoms while others seem to compensate successfully. Both possible components of vestibular compensation, restoration of peripheral vestibular function and saccade patterns (1), can be quantified using the video head impulse test [vHIT, (4–6)], a non-invasive test of the high-frequency vestibulo-ocular reflex (VOR) that is widely applicable (7–9).

In the literature, various vHIT parameters have been associated with relevant symptoms in patients with unilateral vestibular hypofunction, e.g., a higher prevalence of (overt) catch-up saccades (10, 11), a higher amplitude of overt saccades (11) or a higher velocity of covert saccades (10). For the parameters VOR gain and prevalence of covert saccades, partially contradictory results have been presented (10–12). Thus, it is not clear which saccade characteristics are most appropriate to assess successful vestibular compensation in patients with unilateral vestibular hypofunction and whether they are independent of the VOR gain.

In this study, we aimed to determine which parameters of the vHIT best predict the presence of symptoms in individuals with unilateral vestibular hypofunction, embedded in a population-based survey. We divided participants with unilateral vestibular hypofunction into two groups, one with vertigo, dizziness, or balance problems in the past 12 months (symptomatic) and one without any of these symptoms in the past 12 months (currently symptom-free). For each group, age and gender, as well as VOR gain, retinal error, and saccade characteristics, were evaluated and compared.

## 2 Methods

### 2.1 Study design and participants

Data originate from the Cooperative Health Research in the Region of Augsburg (KORA) FF4 study, the second follow-up of the KORA S4 population-based health survey, conducted in 2013/2014 with 2,279 participants [mean age 60.8 years, range 39–88 years, 51.6% female; for more details see (13)]. For an overview of the selection of the analyzed subpopulation, see the flowchart in Supplementary Figure S1. Participants who reported moderate or severe vertigo or dizziness within the past 12 months in a face-to-face interview were invited to undergo a same-day video head impulse test (*n* = 570). A total of 233 currently symptom-free participants, representative of the study population, were planned as controls; 142 participants were not eligible for vHIT due to cervical spine problems. In total, vHIT data from 661 participants were evaluated (see flowchart in the Supplementary material). Applying strict data quality criteria (see Data analysis section), 189 participants had to be excluded from further analysis. Vestibular hypofunction was defined as instantaneous VOR gain at 60 ms after the onset of the head impulse <0.80 [analogous to (10)] and with catch-up saccades present. VHIT data were evaluated by two experienced neuro-otologists (MA, >5 years of experience, and NL, >10 years of experience) and categorized as ‘no vestibular hypofunction’ (*n* = 424), ‘unilateral vestibular hypofunction’ (*n* = 33), or ‘bilateral vestibular hypofunction’ (*n* = 15). Raters were blinded to symptom status. Due to low symptom variability in the bilateral vestibular hypofunction subpopulation (14 symptomatic participants), only the unilateral vestibular hypofunction subpopulation (*n* = 33) was further analyzed.

### 2.2 Measures: outcome

To determine whether participants were classified as symptomatic or currently symptom-free, two interview questions were combined: If the answer to the question ‘Have you ever experienced moderate to severe vertigo, dizziness or balance problems?’ was ‘No’, participants were classified as currently symptom-free, and no further questions were asked about vertigo or dizziness. If the answer was ‘Yes’, a second question was asked: ‘Have you experienced moderate to severe vertigo, dizziness or balance problems in the past 12 months?’. Participants who answered ‘No’ to this question were also classified as currently symptom-free, and those who answered ‘Yes’ were classified as symptomatic (see flowchart in Supplementary Figure S1).

### 2.3 Measures: video head impulse testing (vHIT)

Video head impulse testing was performed using the EyeSeeCam® system. The participant was seated and fixated on a point at eye level on a wall 2 meters in front of them. The examiner, standing behind the participant, applied horizontal head impulses via the jaw (25° head-down position, targeted velocity 150–250°/s, targeted amplitude 6–12°, targeted number of impulses 10–15 to each side). Three examiners with no previous experience in head impulse testing were trained before and during the study and achieved a satisfactory level of quality (14).

The mean number of vHITs in the subpopulation with unilateral vestibular hypofunction was 18 ± 7 per participant per side (right vHITs 17 ± 6 and left vHITs 19 ± 9, mean ± SD). VHIT duration was 136 ± 16 ms (mean ± SD of the median per participant) and covered 12 ± 3° of horizontal rotation with a peak velocity of 175 ± 10°/s. Gains on the vestibular hypofunction side were 0.72 ± 0.10 (mean ± SD of the median per participant), gains on the healthy side were 0.92 ± 0.07.

### 2.4 Measures: covariates

We analyzed the effect of age (at the time of examination), gender, and the following parameters calculated from vHIT data on the presence of symptoms: amplitude of the first catch-up saccade in degrees, latency of the first catch-up saccade in milliseconds, number of catch-up saccades per trial, percentage of covert saccades, instantaneous VOR gain, and retinal error in degrees at 200 ms after the onset of the head impulse. More details on these covariates can be found in the Data analysis section.

### 2.5 Data analysis

Data were analyzed offline using the EyeSeeCam® software to calculate head impulse onset and head and eye velocity; MATLAB® software (MathWorks, Natick, MA) was used for further processing. Impulse traces with peak head velocity slower than 150°/s were automatically discarded, traces with artifacts were discarded after visual inspection (manual correction), according to (14) and (15), including blinks, which have been associated with a higher number of saccades (16). Participants were further analyzed if more than five vHIT trials per side were acceptable.

VOR gain was calculated as the ratio of the median of eye and head velocity of the accepted trials in a window between 55 ms and 65 ms after the onset of the head impulse (instantaneous VOR gain at 60 ms). Possible side-to-side differences (17) were not analyzed.

To distinguish VOR slow and fast phases, a previously described algorithm for separating slow and fast eye movement components during smooth pursuit (18) was adapted to the vHIT. The resulting fast phases were classified as saccadic or non-saccadic by judging whether their amplitude, duration, and peak velocity corresponded to the main sequence relationships of saccades (19). For participants with unilateral vestibular hypofunction, the five parameters that follow were calculated per side and per participant for the affected side. The mean percentage of trials with catch-up saccades (used to calculate the parameters latency and amplitude) in this subpopulation was 94 ± 10% (mean ± SD).

#### 2.5.1 Retinal error

Retinal error was calculated as the median across trials at 200 ms after the onset of the head impulse. This parameter was chosen to assess whether the first 200 ms of the head impulse were critical for the presence of symptoms. It was calculated as the absolute value of the sum of the head and eye positions at 200 ms after the onset of the head impulse, in analogy to (20). The head and eye positions at this point were calculated as the sums of the head and eye velocity samples for a duration of 200 ms from the onset of the head impulse and finally divided by the sampling rate.

The retinal error at 200 ms is influenced by the two main components of head movement compensation, VOR gain and catch-up saccades, and provides an indication of where the visual target is projected on the retina relative to the fovea at that time. When the retinal error is close to zero, the visual target should be close to the fovea, resulting in blur-free vision. In the literature, the threshold for retinal error is set at approximately 0.5 degrees from the center of the fovea to ensure good visual acuity (21). The retinal error was evaluated at 200 ms, an arbitrary point, because after this time, head movement has normally stopped (in our subpopulation, all mean head movements had stopped by 200 ms) and a large proportion of the first catch-up saccades have occurred. We chose a fixed time point to better be able to compare the data.

#### 2.5.2 Number of catch-up saccades

The number of catch-up saccades per trial was calculated as the median across trials. Catch-up saccades were defined as saccades in the direction contralateral to, and thus compensating for, the head movement. Catch-up saccades were considered if they started in a time range from the onset of the head impulse to 700 ms.

#### 2.5.3 Percentage with covert catch-up saccades

For the percentage of trials with covert catch-up saccades, all trials were considered. A catch-up saccade was considered covert if it started within the time of the head movement.

#### 2.5.4 Latency of the first catch-up saccade

The latency of the first catch-up saccade was calculated as the median across trials. Only trials with at least one catch-up saccade were included.

#### 2.5.5 Amplitude of the first catch-up saccade

The amplitude of the first catch-up saccade was calculated as the median across trials. Only trials with at least one catch-up saccade were included.

### 2.6 Statistical analysis

We report absolute and relative frequencies for categorical data and mean with standard deviation for continuous data. Normality was assessed visually using quantile-quantile plots. Group differences were assessed using an independent two-sample Mann–Whitney *U* test for continuous variables and Fishers Exact test for categorical variables.

Multivariate logistic regression was used to investigate the effect of age, gender, and vHIT parameters (predictors) on the presence of symptoms. We report the exponential of each coefficient of the logistic regression exp(b). Exp(b) represents the predicted change in odds for a unit increase in the predictor. An exp(b) of less than one corresponds to decreasing odds of symptom occurrence for increasing values of the predictor; an exp(b) of greater than one corresponds to an increase of the respective predictor being associated with increased odds of symptom occurrence. Stepwise logistic regression based on Akaike Information Criteria (AIC) was used for model selection. The AIC is a measure of model goodness of fit that takes into account the simplicity of a model by penalizing each additional parameter in the model. Given a set of models based on the same data, the best model is the one with the lowest AIC value. We calculated the generalized variance inflation factor (GVIF) to check for multicollinearity among the predictor variables (22). A GVIF greater than five was considered problematic.

All calculations were performed using R Statistical Software (v4.1.2; R Core Team 2021). The two-tailed significance level was set at 5%. Due to the exploratory nature of the study, correction for multiple testing was not performed.

## 3 Results

**Figure 1:**
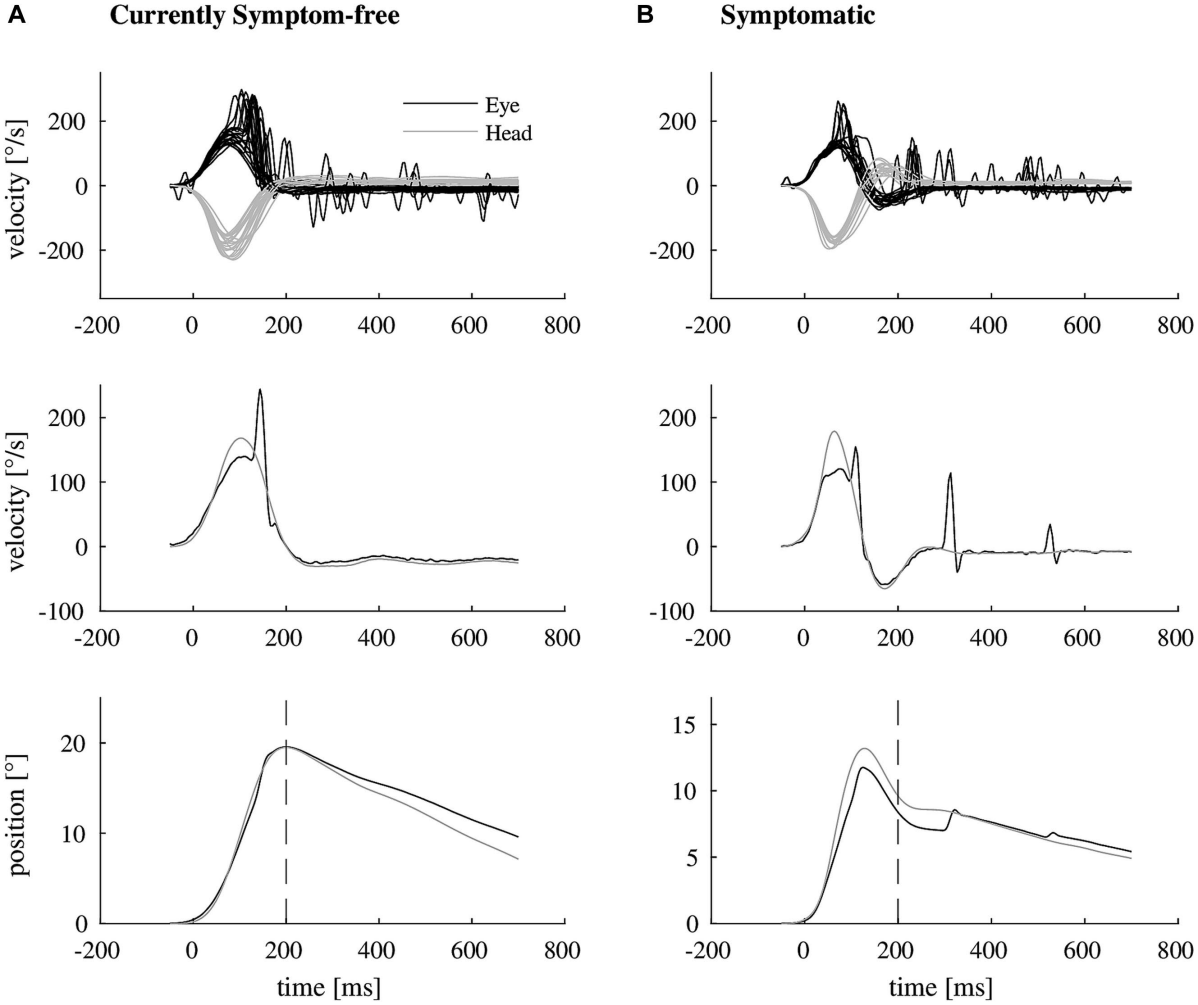
Exemplary video head impulse test data with illustrated retinal error of two participants with unilateral vestibular hypofunction. **(A)** Shows a video head impulse test of a currently symptom-free participant (top, eye movement in black, head movement in gray). The vestibulo-ocular reflex deficit is mostly compensated by one covert catch-up saccade as shown in representative traces of eye and head velocity (middle). As a result, there remains almost no mismatch between eye and head position at 200 ms after the onset of the head impulse (bottom, dashed line). **(B)** Shows the corresponding video head impulse test data of a symptomatic participant (top), needing more than one catch-up saccade for compensation (representative traces in the middle), resulting in a larger retinal error at 200 ms (bottom).

**Figure 2:**
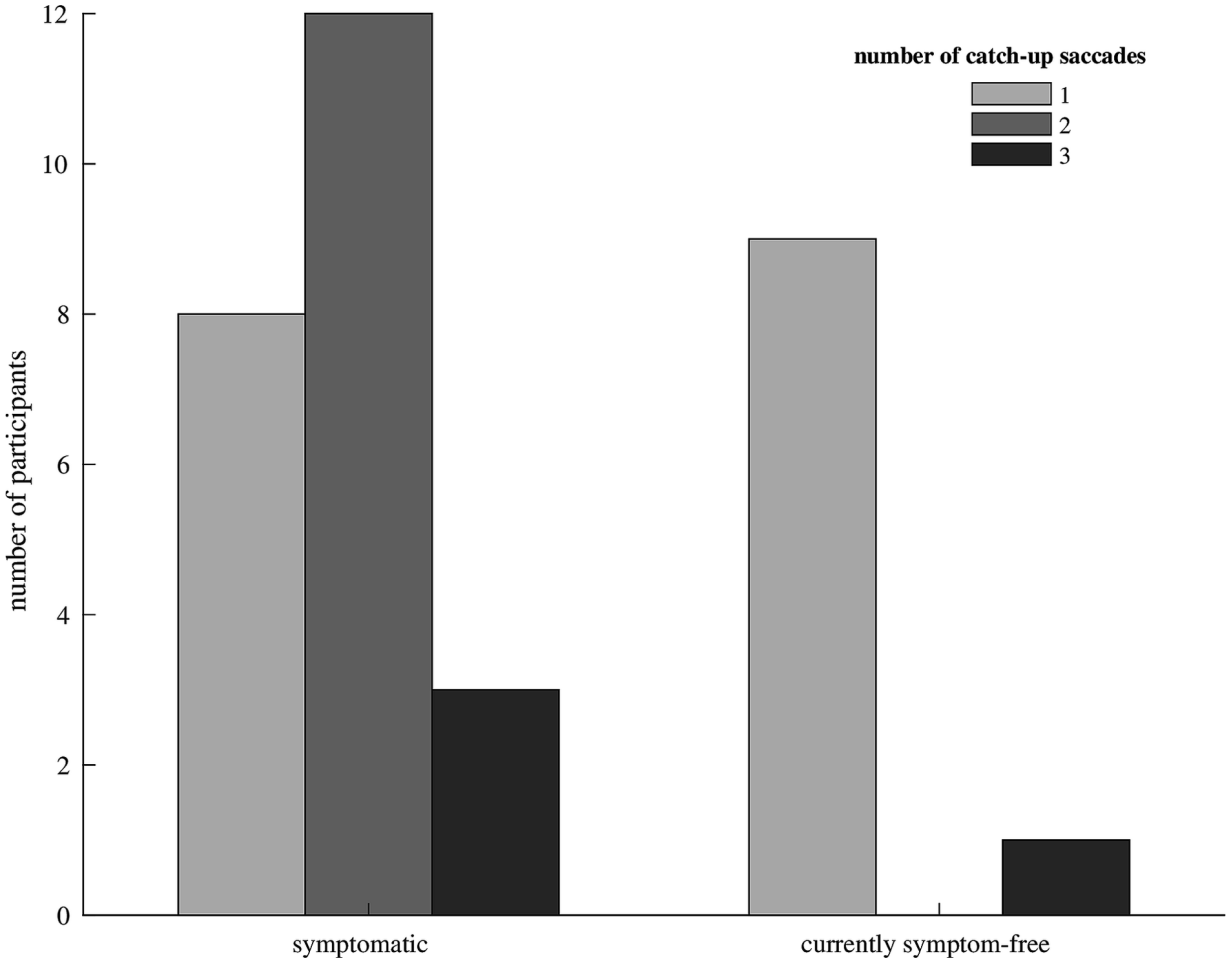
Bar diagram showing the median number of catch-up saccades in participants with symptomatic (left) and currently symptom-free (right) unilateral vestibular hypofunction. Only one currently symptom-free participant showed a median of three catch-up saccades to compensate for the vestibulo-ocular reflex deficit, the remaining currently symptom-free participants needed only one catch-up saccade. Symptomatic participants mostly made two or three catch-up saccades.

We included 33 participants with unilateral vestibular hypofunction with mean age of 68 years (SD = 11); 58% of the participants were female. Participants were categorized as symptomatic (70%) or currently symptom-free (30%). Differences in age or gender between the two groups were not significant (*p* = 0.799 and *p* = 0.057, respectively). Exemplary vHIT traces of a symptomatic participant, showing a higher number of catch-up saccades and a larger retinal error at 200 ms than a currently symptom-free participant with unilateral vestibular hypofunction, are shown in Figure 1. As a group, symptomatic participants showed a significantly larger retinal error at 200 ms after the onset of the head impulse (2.15° vs. 1.24°, *p* = 0.038), a significantly higher percentage of covert saccades (28% vs. 13%, *p* = 0.046), and a significantly higher number of catch-up saccades per trial (1.78 vs. 1.20, *p* = 0.028, bar plot see Figure 2). For the other candidate variables, saccade amplitude, saccade latency and VOR gain at 60 ms, no significant differences were found (see Table 1).

**Table 1:** Mean values with standard deviation (or percentages) and *p*-value (Mann–Whitney-*U* Test or Fishers Exact Test) for each investigated parameter.

Variables	Mean (SD)			*p-*value
	All (*n* = 33)	Symptomatic (*n* = 23)	Currently symptom-free (*n* = 10)	
Number of catch-up saccades	1.61 (0.70)	1.78 (0.67)	1.20 (0.63)	0.013
Retinal error at 200 ms [°]	1.88 (1.24)	2.15 (1.30)	1.24 (0.86)	0.038
Percentage with covert saccades	0.23 (0.29)	0.28 (0.30)	0.13 (0.27)	0.046
Female	0.58*	0.70*	0.30*	0.057**
First saccade latency [ms]	221 (84)	205 (66)	257 (112)	0.264
First saccade amplitude [°]	2.13 (1.27)	2.33 (1.42)	1.68 (0.67)	0.269
VOR gain	0.72 (0.10)	0.72 (0.11)	0.74 (0.07)	0.603
Age [years]	68 (11)	68 (11)	67 (12)	0.799

SD, standard deviation; VOR, vestibulo-ocular reflex.

*Percentage instead of mean; **Fishers exact test instead of Mann–Whitney-*U* test.

Participants with unilateral vestibular hypofunction were classified as symptomatic or currently symptom-free.

Due to multicollinearity (GVIF>5), the variable percentage of covert saccades had to be excluded from the logistic regression model. After stepwise selection based on the AIC, the number of catch-up saccades and the retinal error at 200 ms remained in the final logistic regression model (Table 2). The final model had the lowest AIC (AIC = 37.99), which was significantly better than a null model (*p* = 0.014). The odds for the presence of symptoms increased with a higher number of catch-up saccades (exp(b) = 4.35, 95% confidence interval = [0.98,32.72]) and a larger retinal error at 200 ms after the onset of the head impulse (exp(b) = 1.94, 95% confidence interval = [0.89,5.92]). To give an example, each increase in the number of saccades increased the odds of being symptomatic by 4.35; similarly for retinal error, each point increase increased the odds for being symptomatic by 1.94.

**Table 2:** Results of the logistic regression model for the association between the presence of symptoms and the investigated parameters in participants with unilateral vestibular hypofunction, after applying stepwise variable selection based on the AIC (Akaike Information Criteria, AIC = 37.899).

Variables	exp(b)	95% Confidence interval	*p-*value
Number of catch-up saccades	4.35	[0.98; 32.72]	0.0889
Retinal error at 200 ms	1.94	[0.89;5.92]	0.1655

Exp(b), exponential of each coefficient of the logistic regression.

The investigated parameters were as follows: number of catch-up saccades, retinal error at 200 ms, gender, first saccade latency, first saccade amplitude, vestibulo-ocular reflex gain, and age. We report the exponential of the resulting coefficients together with the 95% confidence interval and the *p*-values. Due to multicollinearity the variable percentage with covert saccades had to be excluded from the logistic regression model.

## 4 Discussion

The number of catch-up saccades and retinal error at 200 ms after the onset of the head impulse best predicted the presence of symptoms in a subpopulation of 33 participants with unilateral vestibular hypofunction in a population-based survey. VOR gain, saccade amplitude, saccade latency, gender, or age did not contribute to discriminating symptomatic from currently symptom-free participants with unilateral vestibular hypofunction. Covert catch-up saccades were more frequent in symptomatic participants, but the overall frequency was less than 30%, and they had to be excluded from the logistic regression model due to multicollinearity.

The fact that the retinal error at 200 ms is predictive confirms that a larger ‘mismatch’ between eye and head position (retinal error) due to a VOR deficit contributes to vertigo, dizziness, or balance problems. This neurophysiological measure, which correlates with presence of symptoms, appears to be consistent with a smaller eye position error during optotype presentation (from 62 ms to 142 ms after the onset of the head impulse) being associated with better dynamic visual performance in patients with unilateral vestibular loss (23). Since the visual and the vestibular systems are available for compensation at 200 ms after the onset of the head impulse (24), our result suggests that these systems may not compensate sufficiently in our subpopulation when participants complain of symptoms. A possible explanation is that both systems are capable of initiating saccades but are not compensating accurately enough. Covert catch-up saccades may occur too early to be visually generated, probably requiring vestibular information (25). To minimize retinal error, the vestibular system must pre-program the saccade at the onset of the head movement. As an open-loop system, since online visual feedback would be too slow, the calibration factor for saccade programming may be responsible for individual differences in saccadic accuracy. Consistent with this interpretation are study results suggesting that vestibular catch-up saccades reduce the retinal position error by only 37% on average (25). In such a case, a second, more visually triggered, and therefore presumably more accurate catch-up saccade, would be required to correct for the remaining retinal error (26). Since any further saccade can only be made after a refractory period, the relevant retinal error would persist for a longer period of time, probably making the presence of symptoms more likely (27). Cases with an inaccurate covert saccade and a correcting overt saccade might present symptoms more often than cases with an accurate early overt saccade. This could explain why symptomatic participants in our study showed a higher number of catch-up saccades and a higher percentage of covert saccades. However, the number of catch-up saccades and the retinal error remained in the final logistic regression model, whereas the relative frequency of covert saccades did not. This suggests that the accuracy of the first saccade in relation to the retinal error during the head impulse may be more important for presence of symptoms than whether it is covert or overt.

The subpopulation in our study had relatively high VOR gains (Table 1). This could be a possible explanation for the relatively low percentage of covert saccades (28), probably being generated by the vestibular system. The relatively high VOR gains may also be responsible for the fact that saccadic accuracy may carry more weight in relation to the presence of symptoms than the VOR gain deficit. This argument may be supported by previous results showing that the presence of symptoms was associated with VOR gain in study populations with mean VOR gain <0.7 (10, 11), but no such association was found in a study population with mean VOR gain of 0.77 (12).

Our study may have implications for rehabilitation strategy. Since our results indicate that saccadic accuracy is relevant, it may be advisable to emphasize gaze stabilization therapies that improve saccadic accuracy (29) in vestibular rehabilitation, probably through integrated oculomotor training. Saccadic accuracy may be a reasonable measure to assess patients with unilateral vestibular hypofunction during vestibular rehabilitation. The number of catch-up saccades may be a more feasible outcome measure, as shown in patients with residual disability after acute unilateral vestibulopathy, where clinical improvement after vestibular rehabilitation was associated with a reduced number of overt catch-up saccades (30). It would be of interest if accurate saccades appear temporarily grouped, e.g., measurable by the Perez Rey (PR) score (31). For future studies, it would be interesting to understand why some patients are likely to be able to produce more accurate saccades than others and how vestibular rehabilitation can best support this.

### 4.1 Study strengths and limitations

Because our data come from a large population-based survey, the results may be more representative than results from laboratory studies or specialized centers. For practical reasons, embedded in a large survey, we analyzed only the horizontal semicircular canals. The number of participants with unilateral vestibular hypofunction was limited, and there was no information on how acute the symptoms were at the time of examination (other than being present in the past 12 months). The vestibular deficits were mostly small, which probably reflects their distribution in the population, but is unlikely to be representative of patients in a clinical setting. Due to the exploratory nature of the study, the results need to be validated in future studies.

## Data availability statement

The raw data supporting the conclusions of this article will be made available by the authors, without undue reservation.

## Ethics statement

The studies involving humans were approved by the Bavarian Chamber of Physicians, Munich (FF4: EC No. 06068). The studies were conducted in accordance with the local legislation and institutional requirements. The participants provided their written informed consent to participate in this study.

## Author contributions

MA: Conceptualization, Formal analysis, Software, Supervision, Writing – original draft, Writing – review & editing. EG: Conceptualization, Writing – review & editing. TE: Formal analysis, Software, Writing – review & editing. ES: Conceptualization, Writing – review & editing. RS: Formal analysis, Writing – review & editing. KJ: Conceptualization, Writing - review & editing. MM: Data curation, Writing – review & editing. RH: Data curation, Writing – review & editing. BL: Data curation, Writing – review & editing. MH: Data curation, Writing – review & editing. K-HL: Data curation, Writing – review & editing. NL: Conceptualization, Formal analysis, Supervision, Writing – review & editing.
