# Supplementary material for: Symptoms in unilateral vestibular hypofunction are associated with number of catch-up saccades and retinal error: results from the population-based KORA FF4 study
Source: Front Neurol. 2023 Nov 28;14:1292312. doi: 10.3389/fneur.2023.1292312 (PMC10715252; doi:10.3389/fneur.2023.1292312)
Supplement: Supplementary file 1 [file Data_Sheet_1.pdf]

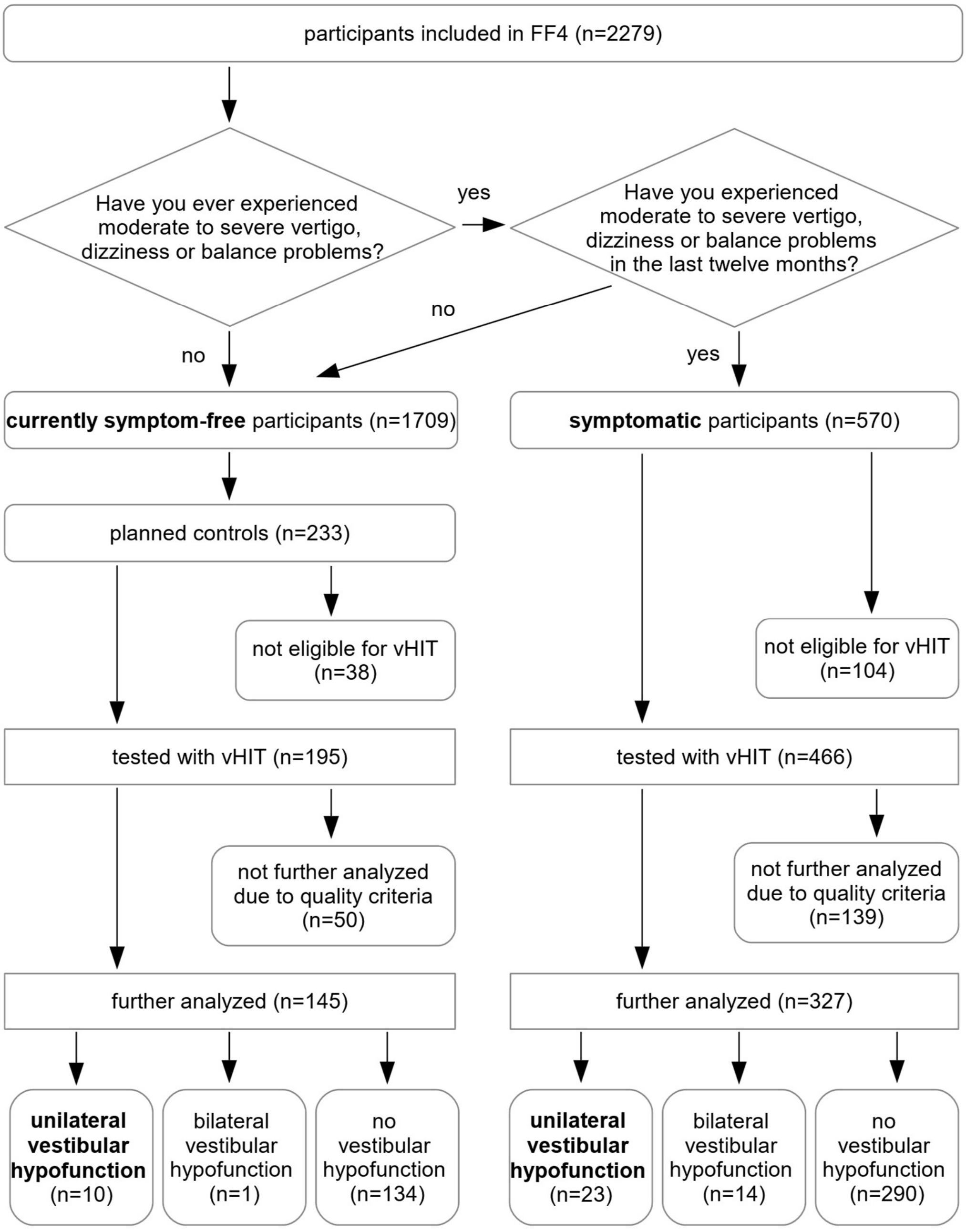

Supplementary Figure S1. Flowchart showing a synopsis of the selection process resulting in the further analyzed subpopulation of 33 participants with unilateral vestibular hypofunction. The 2,279 participants of the Cooperative Health Research in the Region of Augsburg (KORA) FF4 study, the second follow-up of the KORA S4 population-based health survey, were grouped as symptomatic or currently symptom-free according to interview questions. The video head impulse test (vHIT) was not eligible for participants having problems with their cervical spine. Quality criteria are defined in the Data Analysis section.
